# Chronic alcohol ingestion delays skeletal muscle regeneration following injury

**DOI:** 10.1186/2050-490X-1-2

**Published:** 2013-10-01

**Authors:** Graham J Dekeyser, Caroline R Clary, Jeffrey S Otis

**Affiliations:** Currently in Medical School at Georgia Medical College, Georgia, USA; Currently in Medical School at Mercer University, Mercer, USA; Department of Kinesiology and Health, Georgia State University, Atlanta, GA 30322 USA

**Keywords:** Alcoholic myopathy, Skeletal muscle regeneration, Oxidant stress, Glutathione, Fibrosis

## Abstract

**Background:**

Chronic alcohol ingestion may cause severe biochemical and pathophysiological derangements to skeletal muscle. Unfortunately, these alcohol-induced events may also prime skeletal muscle for worsened, delayed, or possibly incomplete repair following acute injury. As alcoholics may be at increased risk for skeletal muscle injury, our goals were to identify the effects of chronic alcohol ingestion on components of skeletal muscle regeneration. To accomplish this, age- and gender-matched C57Bl/6 mice were provided normal drinking water or water that contained 20% alcohol (v/v) for 18–20 wk. Subgroups of mice were injected with a 1.2% barium chloride (BaCl_2_) solution into the tibialis anterior (TA) muscle to initiate degeneration and regeneration processes. Body weights and voluntary wheel running distances were recorded during the course of recovery. Muscles were harvested at 2, 7 or 14 days post-injection and assessed for markers of inflammation and oxidant stress, fiber cross-sectional areas, levels of growth and fibrotic factors, and fibrosis.

**Results:**

Body weights of injured, alcohol-fed mice were reduced during the first week of recovery. These mice also ran significantly shorter distances over the two weeks following injury compared to uninjured, alcoholics. Injured TA muscles from alcohol-fed mice had increased TNFα and IL6 gene levels compared to controls 2 days after injury. Total protein oxidant stress and alterations to glutathione homeostasis were also evident at 7 and 14 days after injury. Ciliary neurotrophic factor (CNTF) induction was delayed in injured muscles from alcohol-fed mice which may explain, in part, why fiber cross-sectional area failed to normalize 14 days following injury. Gene levels of TGFβ_1_ were induced early following injury before normalizing in muscle from alcohol-fed mice compared to controls. However, TGFβ_1_ protein content was consistently elevated in injured muscle regardless of diet. Fibrosis was increased in injured, muscle from alcohol-fed mice at 7 and 14 days of recovery compared to injured controls.

**Conclusions:**

Chronic alcohol ingestion appears to delay the normal regenerative response following significant skeletal muscle injury. This is evidenced by reduced cross-sectional areas of regenerated fibers, increased fibrosis, and altered temporal expression of well-described growth and fibrotic factors.

## Background

Severe insults to skeletal muscle such as blunt force contusions, lacerations, or eccentric contraction overloading may produce extensive injuries that disrupt normal fiber and fascicle architecture while causing temporary weakness [1, 2]. Injured skeletal muscle undergoes several overlapping and well-described phases of healing that include inflammation, necrosis and degeneration, satellite cell activation and proliferation and subsequent regeneration, and scar tissue activation with resultant fibrosis [2–4]. In general, this recovery process is painful and the associated swelling may limit the range of motion near the site of injury. Fortunately, full recovery is often achievable in *otherwise healthy individuals*[1]. In contrast, recent evidence has suggested that pre-existing metabolic conditions such as diabetes mellitus or selenoprotein deficiency may not only affect basal skeletal muscle health, but also the capability of skeletal muscle to regenerate adequately from trauma [5, 6].

We and others have shown that underlying chronic alcohol abuse may produce a wide range of skeletal muscle defects including type II muscle atrophy, oxidant stress, and anabolic resistance [7–11]; however the ability of alcoholic skeletal muscle to regenerate following injury has not been investigated. This issue is clinically relevant as alcoholics may be at increased risk for muscle injuries due in part to peripheral neuropathies and sensory impairments, increased risky behaviors that may include motor vehicle accidents or inter- and intra-personal violence, or to muscle atrophy with concomitant reductions to strength and reduced ability to safely stabilize the body during a fall [12, 13]. Further, understanding the pathophysiological response of muscle from alcohol-fed mice to injury is necessary to develop effective treatments to improve regeneration.

For example, research has shown that *de novo* protein synthesis and accretion play central roles in the repair process [2]. However, muscle from alcohol-fed mice is more resistant to common anabolic stimulation due in large part to altered IGF-1 signaling, impaired protein translation, and nutrient deficiencies[8, 14–16], which may confound the ability of injured muscle from alcohol-fed mice to sufficiently synthesize proteins during repair. In parallel, alcoholic skeletal muscle is marked by elevated oxidant stress, glutathione depletion, elevated levels of catabolic factors, and increased apoptosis [7, 8, 17, 18] which may further impede the regeneration process. The notion of altered regeneration in muscle from alcohol-fed mice is supported by data from cell culture models of myogenesis. For example, culturing skeletal muscle cells in physiological levels of ethanol reduced their cellular proliferation and subsequently delayed their differentiation [19, 20]. Further, protein synthesis is decreased in human muscle cells grown in ethanol-containing media. These cells also have reduced capacity to minimize proteolysis following direct IGF-1 or insulin stimulation [21].

Based on these whole animal and cell culture data, we hypothesized that muscle from alcohol-fed mice may be primed for delayed or incomplete regeneration following significant injury. In this report, we analyzed the influence 18 weeks of chronic alcohol ingestion had on mouse skeletal muscle regeneration following barium chloride (BaCl_2_)-induced tibialis anterior injury. Because chronic alcohol abuse is an addiction and likely to persist following trauma, we chose to analyze the regenerative response in the presence of continued alcohol consumption over the subsequent 14 days of recovery.

## Results and discussion

### Results

#### *Body weight and voluntary wheel running activity*

To indirectly assess the severity of muscle injury, we recorded changes to body weight and quantified daily and total, 14-day voluntary wheel running activity in control and alcohol-fed mice. Pre-injury body weights were similar in control-fed or alcohol-fed mice suggesting that caloric intake did not differ between groups (Table 1). Likewise, there was no effect of muscle injury on body weight in control-fed mice suggesting that muscle injury did not affect normal feeding or drinking patterns. In contrast, body weights of injured, alcohol-fed mice were significantly decreased up to 7 days post-injury compared to uninjured alcohol-fed mice. However, injured alcohol-fed mice returned to their pre-injury body weight after 14 days of recovery.

**Table 1:** **Changes to body weight of alcoholic and control mice following muscle injury**

	Body weights (g)			
	Pre-injury	Post-injury day 2	Post-injury day 7	Post-injury day 14
**Control**	26.9 + 0.3	26.5 + 0.6	28.6 + 0.3	27.1 + 0.3
**Ethanol**	27.4 + 0.4	24.9 + 0.4 #	25.7 + 0.3 *, #	26.6 + 0.1

Alcohol-fed mice experienced a significant decrease in body weight at 2 and 7 days following muscle injury – an effect not apparent in injured, control-fed mice. However, injured alcohol-fed mice returned to their pre-injury body weight after 14 days of recovery from BaCl_2_ injection. Significance was accepted at p ≤ 0.05. *, compared to control-fed, injured group. #, compared to EtOH-fed, uninjured group.

**Figure 1:**
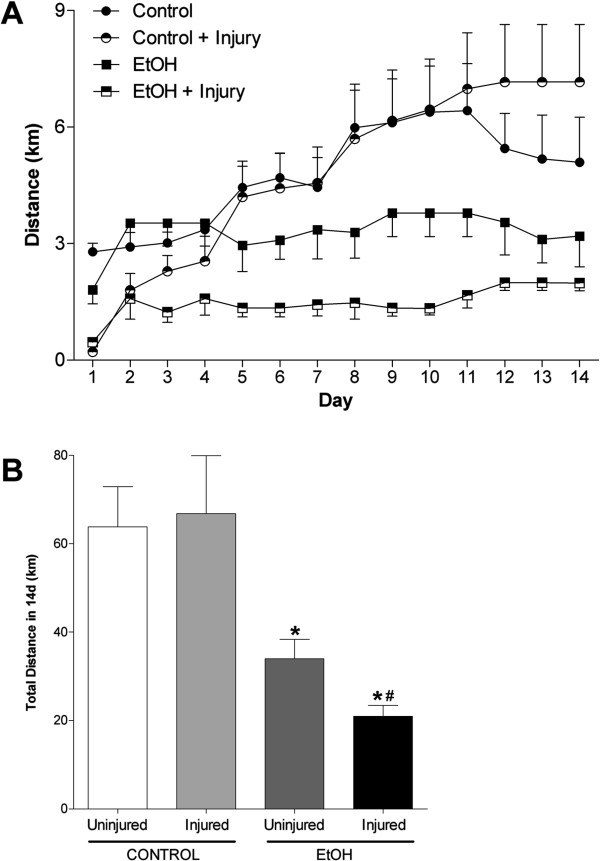
**Chronic alcohol ingestion and muscle injury decreased voluntary running distances.** Injured or uninjured alcoholic and control mice were provided access to a voluntary running wheel. Voluntary activity was measured by **(A)** daily and **(B)** total distance accrued over 14 days. Alcohol-fed mice voluntarily ran ~53% of the distance covered by control-fed mice. In control mice, daily running distances were temporarily reduced following injury (up to day 5 in A); however, total distances in 14 days were similar. In contrast, injured alcohol-fed mice ran significantly less over 14 days compared to uninjured alcoholics. Significance was accepted at p ≤ 0.05. *, compared to control-fed, injured group. #, compared to EtOH-fed, uninjured group.

In addition, we hypothesized that the influence of chronic alcohol ingestion and severity of skeletal muscle injury may affect voluntary wheel running activity. Accordingly, we next measured the daily and total, 14-day distances that control-fed or alcohol-fed mice ran (Figure 1A and B, respectively). Despite an initial delay in voluntary running activity, muscle injury did not affect total 14-day distance in control-fed mice. In the absence of injury, alcohol-fed mice voluntarily ran ~53% of the distance accrued by control-fed mice. However, when alcohol-fed mice were injured their total 14-day distance sharply decreased compared to their uninjured counterparts.

#### *Markers of inflammation and oxidant stress*

**Figure 2:**
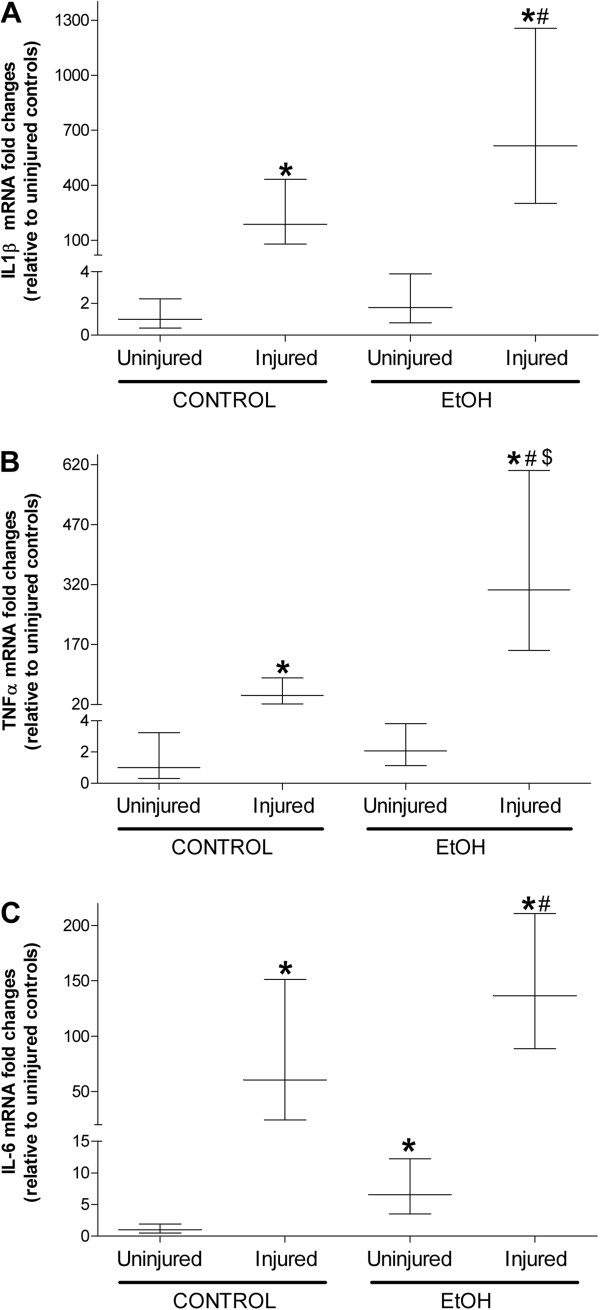
**Injured muscle has a more pronounced inflammatory cytokine response in alcohol-fed mice.** Real time PCR analyses of 3 inflammatory cytokines were performed in tibialis anterior (TA) muscles 2 days after BaCl_2_-induced injury in mice provided alcohol in their drinking water for approximately 16 weeks. Injured TA muscles from control-fed mice had increased gene levels of **(A)** IL1β, **(B)** TNFα, and **(C)** IL6. Likewise, this pattern of increased expression persisted in injured muscles from EtOH-fed mice. Of the three, common inflammatory cytokines associated with early repair processes, gene levels of TNFα were higher in injured muscle from alcohol-fed mice compared to injured controls. Data are represented as means ± range of potential values based on the 2^-∆∆C^_T_ method [7, 8, 22, 25, 48] and expressed as fold changes relative to uninjured controls. Significance was accepted at p ≤ 0.05. *, compared to control-fed, uninjured group. #, compared to EtOH-fed, uninjured group, $ compared to control-fed, injured group.

Next, we determined the co-morbid effect of chronic alcohol ingestion and skeletal muscle injury on inflammatory cytokine gene profiles. Two days following injury, TA muscles from control-fed mice had significantly elevated levels of IL1β, TNFα, and IL6 (Figure 2A-C, respectively). Consistent with our previous results [8], alcohol ingestion drove skeletal muscle expression of IL6 – an effect that was further increased following injury (Figure 2C). Likewise, gene expression levels of TNFα were significantly increased in alcoholic TA muscles two days following injury compared to uninjured muscles (Figure 2B).

**Figure 3:**
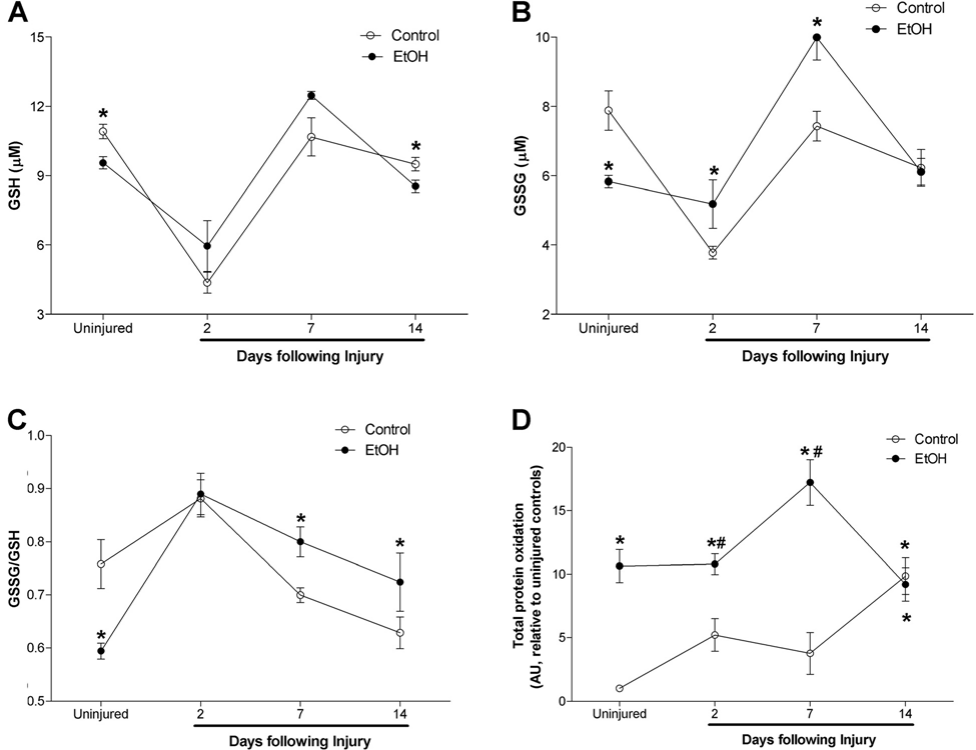
**Oxidant stress is greater in injured muscles from alcohol-fed mice during regeneration.** Glutathione (GSH), oxidized glutathione (GSSG), and total protein oxidation were used as markers of oxidant stress due to chronic alcohol ingestion and muscle injury. **(A)** Levels of GSH were lower in uninjured muscle and in muscle 14 days after injury in alcohol-fed mice. **(B)** Although GSSG levels were lower in uninjured muscle from alcohol-fed mice muscle compared to controls, these levels rose in TA muscles from alcohol-fed at 2 and 7 days following injury before normalizing at day 14. **(C)** The GSSG/GSH ratio, a marker of glutathione status, suggested increased oxidant stress 7 and 14 days after injury in muscle from alcohol-fed mice. **(D)** Total protein oxidation based on carbonyl formation suggested that muscle from alcohol-fed mice is more oxidized basally, and at 2 and 7 days following injury. Significance was accepted at p ≤ 0.05. *, compared to control-fed, uninjured group. #, compared to control-fed, injured group matched to time following injury.

Because chronic alcohol ingestion perturbs glutathione metabolism [7, 8, 22], we next determined the effects of chronic alcohol ingestion and skeletal muscle injury on glutathione (GSH) and oxidized glutathione (GSSG) levels. Similar fluctuations to GSH pools were evident in injured TA muscles from control- and alcohol-fed mice (Figure 3A). However, GSSG levels were significantly elevated in injured, alcoholic TA muscles at 2 and 7 days following injury and returned to baseline levels at 14 days (Figure 3B). Together, the GSSG/GSH ratio – a marker of the oxidative state of the glutathione pool – was increased in alcoholic TA muscles at 7 and 14d following injury (Figure 3C). In addition, we performed dot blot analyses of carbonyl formation to quantify the total amount of protein oxidation in alcoholic, injured skeletal muscle (Figure 3D). Chronic alcohol ingestion alone increased total skeletal muscle protein oxidation compared to controls. Injured, alcoholic TA muscle had elevated total protein oxidation at 2 and 7 days of recovery – an effect that was normalized at 14 days.

#### *Morphology of injured muscles from alcohol-fed mice*

**Figure 4:**
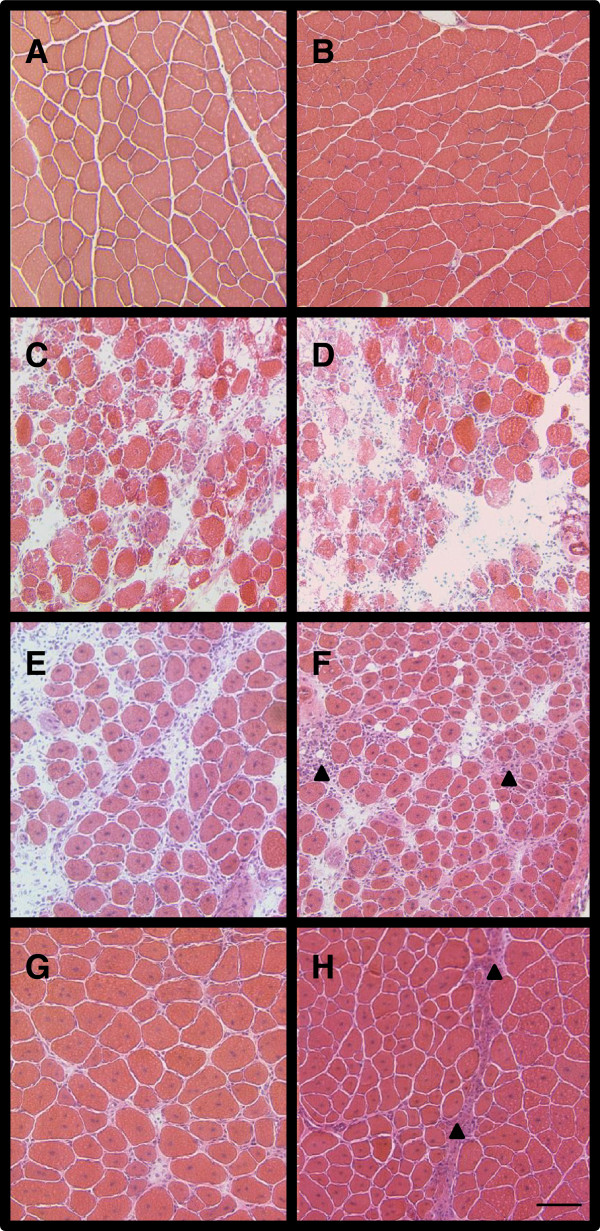
**Representative histology of regenerating TA muscles following injury in alcohol-fed mice.** The processes of skeletal muscle injury and repair in TA muscles from control-fed mice are displayed in panels **(A)** uninjured muscle, and **(C)** 2 days, **(E)** 7 days, and **(G)** 14 days following BaCl_2_ injection. Similarly, muscle regeneration in alcohol-fed mice is displayed in panels **(B)** uninjured muscle, and **(D)** 2 days, **(F)** 7 days, and **(H)** 14 days following BaCl_2_ injection. Arrow heads in panels F and H denote areas of increased interstitial tissue accumulation and fibrosis in regenerating muscle from alcohol-fed mice. Cross-sectional areas of regenerating fibers, as noted by the presence of centralized nuclei shown in panels E-H, have been quantified in Figure 4. Bar in panel H = 100μm.

**Figure 5:**
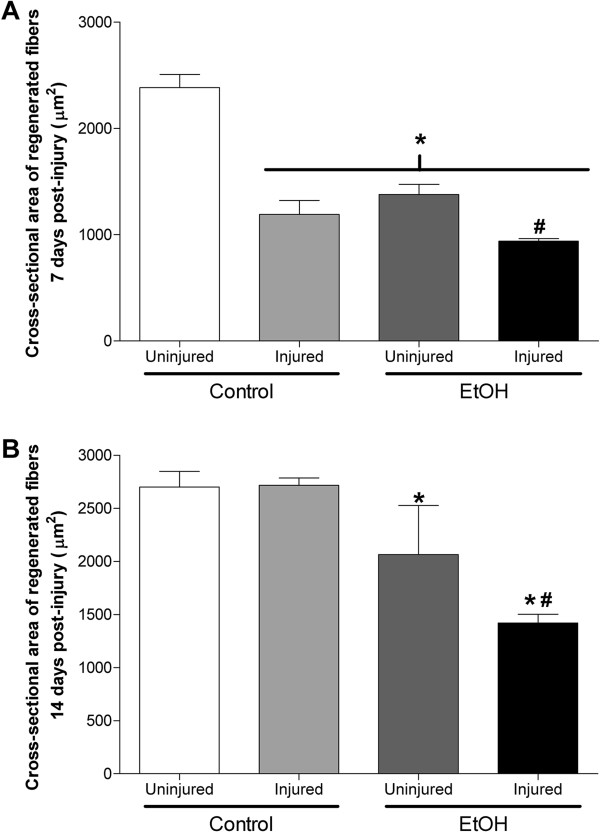
**Recovery of TA fiber area is delayed following muscle injury in alcohol-fed mice. (A)** Regardless of diet, regenerating muscle fibers are smaller than their uninjured, contralateral TA areas. Moreover, regenerating fibers from alcohol-fed mice are smaller than those from alcohol-naïve mice. **(B)** In control-fed mice, regenerating fibers are similar in cross-sectional area as the uninjured, contralateral TA muscles 14 days after injury. In contrast, regenerating fibers from alcohol-fed mice are still smaller than alcoholic, uninjured muscle. This suggests that regeneration in the presence of continued alcohol ingestion delays skeletal muscle repair following injury. Significance was accepted at p ≤ 0.05. *, compared to control-fed, uninjured group. #, compared to EtOH-fed, uninjured group.

We next calculated the cross-sectional areas of regenerating fibers at days 7 and 14 (panels in Figure 4 show representative H&E staining). Areas of regenerating, centrally-nucleated fibers, regardless of diet, are smaller than their contralateral, uninjured controls 7 days following BaCl_2_ injection (Figure 5A). Moreover, regenerating alcoholic fibers are smaller than those from injured control-fed mice. By 14 days, regenerating fibers in control-fed mice are similar in size to those from uninjured muscle (Figure 5B). In contrast, regenerating fibers from alcohol-fed mice are significantly smaller than uninjured, alcoholic fibers suggesting a delay in the normal regenerative response.

#### *Markers of repair and fibrosis*

**Figure 6:**
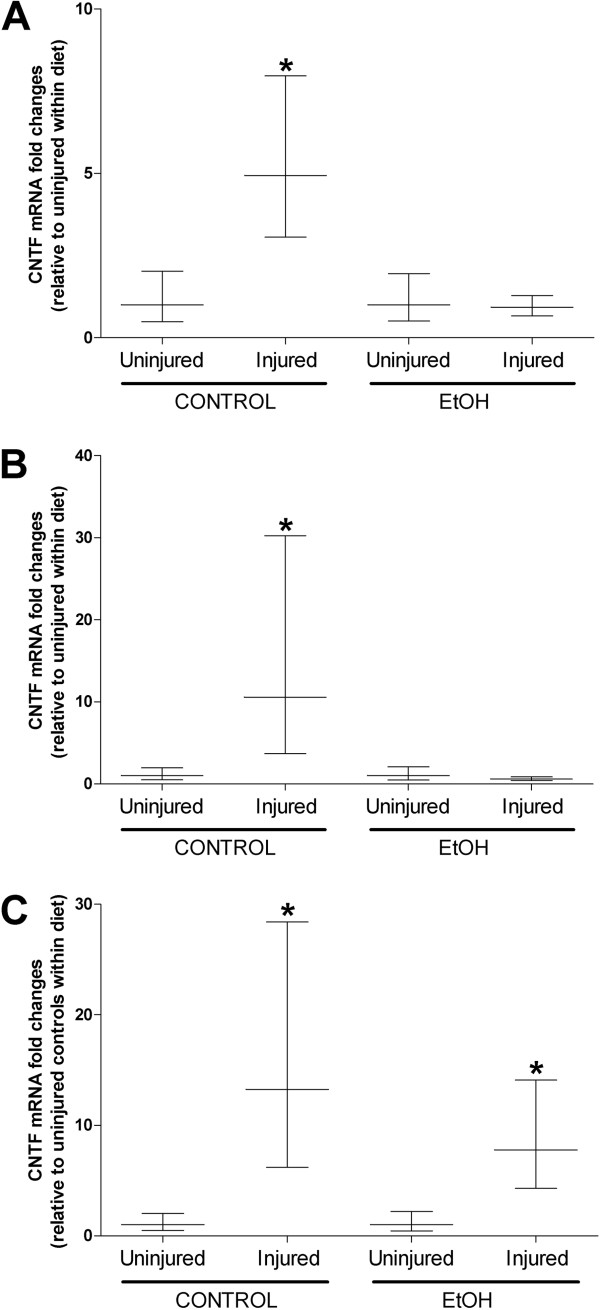
**Delayed CNTF gene expression during muscle recovery from injury may hamper regeneration in alcohol-fed mice.** CNTF, a growth factor implicated in muscle regeneration, was induced at 2, 7, and 14 days following injury in control-fed mice (**A-C**, respectively). However, CNTF was only induced at 14 days following injury in alcohol-fed mice suggesting delayed regeneration. Significance was accepted at p ≤ 0.05. *, compared to uninjured group within diet.

**Figure 7:**
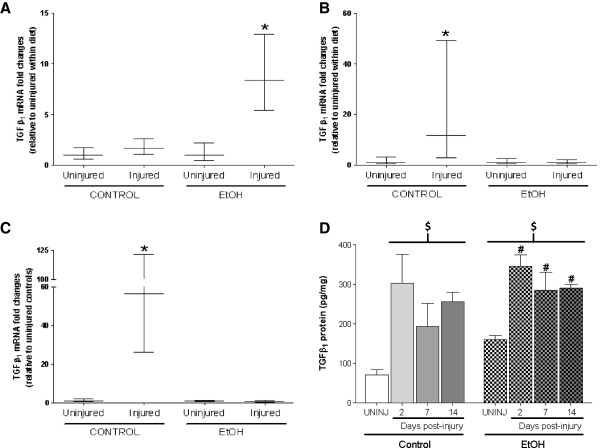
**Chronic alcohol ingestion and injury drive TGFβ expression.** TGFβ_1_, a regulator of skeletal muscle fibrosis, was induced at **(B)** 7 days and remained elevated at **(C)** 14 days following BaCl_2_ injection. **(A)** In contrast, TGFβ_1_ gene levels peaked 2 days following injury in alcohol-fed mice before returning to baseline levels. **(D)** Despite these discrepancies at the gene level, TGFβ protein content was elevated at each time point following injury and likely drives the robust fibrotic response. Significance was accepted at p ≤ 0.05. *, compared to uninjured group within diet. #, compared to EtOH-fed, uninjured group. $, compared to control-fed, uninjured group.

We next identified the co-morbid effects of chronic alcohol ingestion and muscle injury on CNTF gene levels and TGFβ_1_ gene and protein expression levels as these two factors have been implicated in skeletal muscle regeneration and fibrosis, respectively [3, 23, 24] . In order to isolate the effects of muscle injury, gene expression data appearing in Figures 6 and 7 have been normalized to uninjured muscle within diet. Levels of CNTF gene expression were significantly elevated at 2, 7, and 14 days following injury in control muscle (Figure 6A-C, respectively). However, CNTF was not induced in alcoholic TA muscles until 14 days post-injury suggesting a delayed regenerative response (Figure 6C).

We have previously shown that chronic alcohol ingestion alone increased TGFβ_1_ gene expression levels [7, 8, 25] which likely accounts for the increased protein content of the inflammatory cytokine in uninjured muscle (Figure 7D). Despite these elevated basal levels of TGFβ_1,_ significant muscle injury further induced the cytokine after 2 days of recovery - an effect that was not evident until 7 and 14 days of recovery in control-fed animals (Figure 7A-C). However, TGFβ_1_ protein levels were elevated at each time point of recovery regardless of diet (Figure 7D).

**Figure 8:**
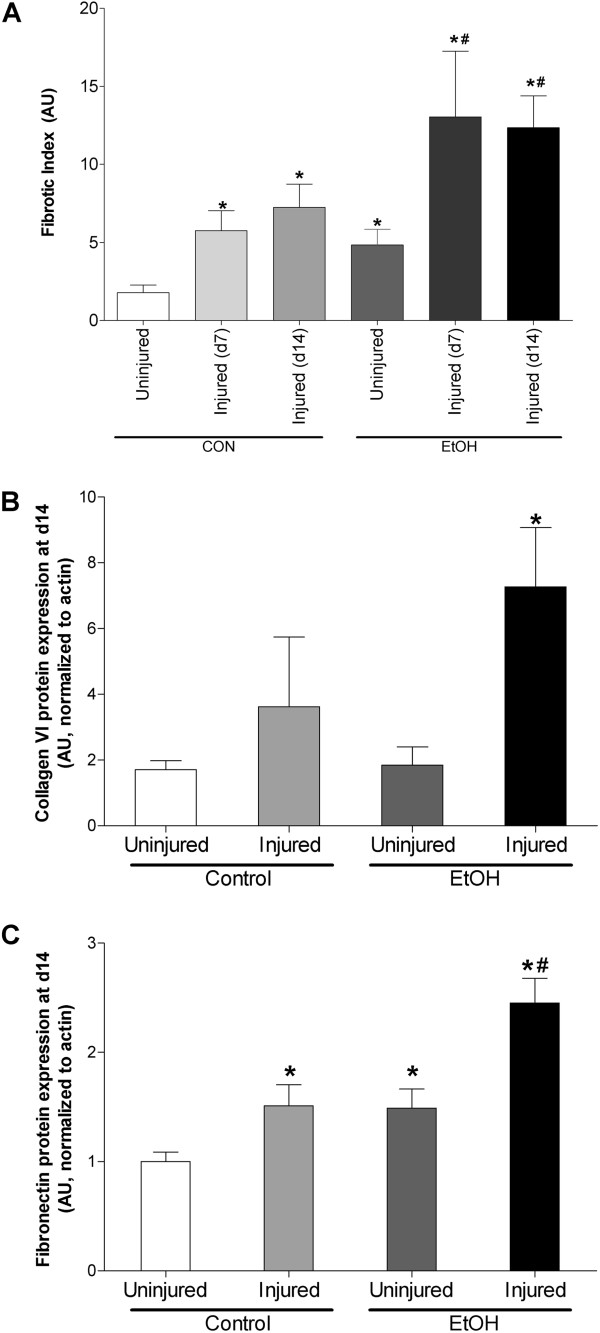
**Markers of fibrosis suggest more pronounced injury in muscle from alcohol-fed mice. (A)** Fibrotic index, a marker of collagen content quantified following trichrome staining, was increased at 7 and 14 days following injury in control-fed mice. Muscle from alcohol-fed mice reported a more robust fibrotic response at these same time points following injury. **(B)** Collagen VI, a component of fibrotic plaques, was increased 14 days after injury in muscle from alcohol-fed mice. **(C)** Fibronectin, an extracellular matrix glycoprotein associated with wound healing, was elevated in injured control muscle 14 days after injury. However, fibronectin expression was further increased in injured muscle from alcohol-fed mice. Significance was accepted at p ≤ 0.05. *, compared to control-fed, uninjured group. #, compared to control-fed, injured group matched to recovery time.

Regenerating muscles from control-fed mice displayed a step-wise increase in the fibrotic index, a measure of collagen density and surrogate marker of overall fibrosis, at 7 and 14 days following injury (Figure 8A). Basally, the fibrotic index of uninjured TA muscle from alcohol-fed mice was higher than controls. Further, the fibrotic index of regenerating muscle from alcohol-fed mice was significantly at both 7 and 14 days following injury compared to time-matched controls (Figure 8A). To verify the fibrotic index, we next measured collagen VI (Figure 8B) and fibronectin (Figure 8C) protein expression in TA muscles 14 days following injury. Both collagen VI and fibronectin levels were significantly elevated in injured muscle from alcohol-fed mice suggesting a more robust fibrotic response compared to controls.

### Discussion

In this study, we determined the effects of chronic alcohol ingestion on distinct components of skeletal muscle regeneration, including the early inflammatory cytokine response, the development of oxidant stress and temporal alterations to glutathione levels, levels of growth and pro-fibrotic factors, regenerated fiber areas, and fibrosis. The co-morbid effects of chronic alcohol ingestion and skeletal muscle injury temporarily reduced body weight and significantly affected voluntary wheel running activity. Further, muscle from alcohol-fed mice had a more robust cytokine response and prolonged duration of oxidant stress due to injury. Induction of ciliary neurotrophic factor (CNTF), a growth factor implicated in myotube differentiation and accelerated muscle repair [26], was postponed in injured muscle from alcohol-fed mice. Similarly, temporal variations existed in the gene expression of the pro-fibrotic cytokine TGFβ_1,_ despite similar inductions at the protein level due to injury. Together, these data may suggest that this robust cytokine response and prolonged duration of oxidant stress when occurring in concert with altered expression patterns of growth and fibrotic factors may, in part, account for the delayed regeneration and increased fibrosis in injured muscle from alcohol-fed mice. Thus, therapeutic strategies that attenuate surges in catabolic factors common to both chronic alcohol ingestion and muscle injury while simultaneously correcting deficits to protein synthesis and fibrosis pathways may improve the regenerative response in injured muscle from alcoholics.

Left untreated, skeletal muscle injuries may result in persistent weakness, decreased flexibility and mobility, or increased likelihood of re-injury. To date, several treatments for mild to severe muscle injuries have been used, including physical modalities such as the R.I.C.E. principle (Rest, Ice, Compression, Elevation) [27],therapeutic ultrasound [28], pharmacologic interventions such as non-steroidal anti-inflammatory drugs (NSAIDS) [29] or administration of growth hormone and IGF-1 [30], or drugs that curb fibrosis by blocking TGFβ_1_ induction with compounds such as suramin [31, 32]. Alternatively, one may consider anti-oxidant supplementation to attenuate oxidative stress-mediated muscle degeneration; however this strategy has been attempted with mixed results [33–35]. For example, N-acetyl cysteine (NAC) and ascorbic acid (Vitamin C) supplementation improved plasma super oxide dismutase and glutathione peroxidase activities, but had little influence on skeletal muscle regeneration in otherwise healthy adults [33]. In contrast, improved muscle repair was achieved using NAC in subjects with underlying chronic disease [34]. For example, Vercherat and colleagues showed that NAC supplementation in Stra13^-/-^ mice, a murine model of dystrophy-like pathology, ameliorated oxidative stress and minimized muscle necrosis. Thus, these results suggest that treating oxidant stress associated with pre-existing pathologies may have the additional benefit of improving functional recovery of degenerating skeletal muscle. This finding is important as we and others have long-described the influence of chronic alcohol ingestion on free radical generation, elevated oxidative stress, glutathione depletion, and induction of redox-sensitive catabolic signaling mechanisms [7, 8, 36–39]. Thus, we believe that appropriate anti-oxidant supplementation may provide a clinically-relevant, convenient treatment option for alcoholic myopathy and act as a protective countermeasure to alleviate acute surges in oxidant stress and redox-sensitive factors following injury. As such, we are currently testing the effectiveness of several dietary glutathione precursors on repair processes in injured, alcoholic muscle.

Specifically, we have previously shown and confirmed here that chronic alcohol ingestion increased TGFβ_1_ levels in skeletal muscle [7, 8]. Alcoholic muscle also had higher basal levels of interstitial fibrosis, a phenomenon recently described in triceps surae muscles from alcoholic rats [40]. Thus, higher basal levels of TGFβ_1_ and fibrosis in alcoholic muscle may predispose the muscle to a more robust fibrotic response following acute injury. Interestingly, TGFβ_1_ protein levels were similarly induced regardless of alcohol status and may suggest that fibrosis in regenerating alcoholic muscle may be regulated partly by other members of the TGFβ superfamily [41]. Ciliary neurotrophic factor (CNTF) is a member of the IL6 family of cytokines and has been shown to accelerate myotube differentiation and regenerated fiber number following exogenous application to injured muscle [26]. CNTF may also play a significant role in muscle repair by stimulating neuronal outgrowth [42] and subsequent re-innervation of damaged fibers [43]. Here, chronic alcohol ingestion delayed the temporal expression pattern of injury-induced CNTF. CNTF did not peak in muscle from alcohol-fed mice until 14 days after injury and its absence during the earlier stages of recovery may have contributed to the faulty regenerative response beyond 14 days of recovery in muscle from alcohol-fed mice.

## Conclusions

We showed that chronic alcohol ingestion altered the regenerative response following skeletal muscle injury. Specifically, areas of regenerated fibers were significantly smaller after 7 and 14 days of recovery from BaCl_2_-induced injury in alcohol-fed mice. In addition, injured muscle from alcohol-fed mice had increased fibrosis compared to controls. These effects were likely mediated, in part, by altered temporal expressions of CNTF and TGFβ_1_, respectively.

## Methods

### Animals and diets

Male C57Bl/6 mice (~ 8 wk) were purchased from The Jackson Laboratories (Bar Harbor, ME), housed in groups of five under a 12:12 light–dark cycle, and provided standard rodent chow ad libitum. All procedures were approved by the Emory University Institute for Animal Care and Use Committee.

Subgroups of mice were provided alcohol-containing drinking water for 18–20 weeks as previously described [44]. Briefly, alcohol was added gradually to acclimatize the mice to the diet and was sequentially added as 5%, 10%, and 15% for 3–4 days each before finally provided as 20% (v/v) for the duration of the experiment. Barium chloride (BaCl_2_)-induced injuries were performed in mice that had been on 20% alcohol for up to 18 weeks. Body weights were recorded before muscle injury. Following muscle injury, mice returned to either control or alcohol diet until the study’s conclusion. At this time, mice were euthanized following CO_2_ asphyxiation, weighed, and their tibialis anterior (TA) muscles were removed, blotted dry, weighed, and prepared for further analyses.

### Barium chloride-induced skeletal muscle injury

Alcoholic or control mice were anesthetized by intraperitoneal injection of a xylazine (15 mg/kg) and ketamine (100 mg/kg) cocktail. Fifty-μl of a 1.2% BaCl_2_ solution were injected into mouse TA muscles using a Hamilton syringe and 27 G needle as previously described [45]. Contralateral TA muscles served as uninjured controls. Briefly, the needle was inserted at the origin of the TA, extended past the mid-belly of the muscle to a region just superior to the distal tendon. Next, the diluted BaCl_2_ solution was continuously injected into the TA as the syringe was removed. Complete serial sections of the injured TA confirmed that the mid-belly section was affected by the myotoxin (unpublished observations). Injured and uninjured, contralateral control muscles were harvested *post mortem* at 2, 7, or 14 days following injury (n = 6-8/time) and processed for markers of degeneration and regeneration.

### Voluntary wheel running

To assess the influence of alcohol and/or injury on voluntary activity, subgroups of injured or uninjured alcoholic and control-fed mice were provided free access to a running wheel (Super pet comfort wheel, 5.5” diameter, PetSmart, Inc., Phoenix, Arizona) that was equipped with a cycle monitor (Velo 8 computer, CatEye Co. LTD, Osaka, Japan). Animals were maintained on either the alcoholic or control diet. Daily (24 h) and total distance accrued over 14 days were recorded.

### Histological assessment of regeneration and fibrosis in injured TA muscles

Uninjured and injured TA muscles were removed, embedded in OCT, and immediately frozen in isopentane cooled in liquid nitrogen. To ensure that we were assessing the most damaged portion of the TA, muscles were cut in serial sections at 10 μm beginning at the mid-belly. Sections were then processed for hematoxylin and eosin staining, dehydrated, mounted, and visualized at 10X with a Leica microscope as previously described [7, 8, 22, 46]. Cross-sectional areas of approximately 200 centrally-nucleated fibers (i.e., regenerated fibers) per muscle were calculated using ImageJ software (NIH, Bethesda, MD).

Fibrosis was determined using trichrome staining as previously described [31]. The fibrotic index was calculated using ImageJ software and represents the density of blue, collagen-stained areas relative to the entire muscle-containing visual field of at least 2 independent fields. Collagen VI and fibronectin western blots were used to complement and confirm data used to calculate the fibrotic index.

### Western blot analyses

Equal amounts of protein were boiled for 2 minutes in sample buffer that contained: 0.5M Tris–HCl (pH 6.8), 10% (v/v) glycerol, 10% (w/v) SDS, 5% (v/v) β-mercaptoethanol, and 0.05% (w/v) bromophenol blue. Samples were separated by SDS-PAGE and transferred onto nitrocellulose membranes using a trans-blot SD semi-dry transfer cell (Biorad, Hercules, CA) as previously described [25]. All incubations were performed at room temperature unless otherwise noted. Membranes were blocked in 5% BSA diluted in TTBS (0.01% (w/v) Tween-20) for 1 h and then incubated in primary antibodies against collagen type VI (Cell Signaling Technology, Beverly, MA; 1:1000 in blocking buffer), fibronectin and actin (Santa Cruz Biotechnology Inc., Santa Cruz, CA; 1:250 in blocking buffer) overnight at 4°C. Blots were washed in TTBS, incubated in anti-rabbit-HRP IgG (1;2500 in blocking buffer) for 1 h, washed again and then developed with enhanced chemiluminescent plus western blotting detection system (GE Healthcare, Piscataway, NJ). Densitometry was performed using a Chemidoc XRS system and analyzed with Quantity One software (Biorad, Hercules, CA). Collagen type VI and fibronectin have been normalized to total actin content.

### TGFβ_1_ immunoassay

TGFβ_1_ protein content was determined in skeletal muscle homogenates using a commercially available ELISA-based assay kit according to manufacturer’s instructions (R&D Systems, Minneapolis, Minnesota). Briefly, frozen TA muscles were washed, minced, and homogenized in ice-cold PBS. Homogenates were centrifuged for 5 min at 5000g and supernatants were used for quantification of total TGFβ_1_ protein content. Levels of TGFβ_1_ were normalized to total protein in the homogenate using standard protein assay techniques [47].

### Real-time polymerase chain reaction (RT-PCR)

TA muscles were immediately frozen in liquid nitrogen and stored at -80°C until processed for RT-PCR analyses as previously described [7, 8, 22, 46]. Briefly, trizol was added (1 ml/100 mg tissue) and the tissues homogenized using an electric tissue homogenizer. Total RNA (2.5 μg) was reverse transcribed in a 25–50 μl final reaction volume using random primers and M-MLV reverse transcriptase (Invitrogen, Carlsbad, CA). The reverse transcription reaction was incubated at 65°C for 10 min, 80°C for 3 min, and 42°C for 60 min. RT-PCR products were analyzed using the iCycler iQ system (Bio-Rad, Hercules, CA). cDNA (5 μl of a 1:10 dilution) was amplified in a 12.5 μl reaction containing 400-nm gene-specific primer pair and iQ Sybr Green Supermix (Bio-Rad). Primers were as follows:IL1β, 5’-AGAGCATCCAGCTTCAAATCTC-3’ and 5’-CAGTTGTCTAATGGGAACGTCA-3’; IL6, 5’-CAAAGCCAGAGTCCTTCAGAG-3’ and 5’-GTCTTGGTCCTTAGCCACTCC-3’; TNFα, 5’-TGGCCCAGACCCTCACACTC-3’ and 5’-CTCCTGGTATGAAATGGCAAATC-3’; and TGFβ_1_, 5’-GAGACGGAATACAGGGCTTTC-3’ and 5’-CAACCCAGGTCCTTCCTAAAG-3’. Samples were incubated at 95°C for 15 min, followed by 40 cycles of denaturation, annealing, and extension at 95°C, 60°C, and 72°C, respectively. As a control, RT-PCR was also performed on 2 μl of each RNA sample to confirm absence of contaminating genomic DNA. Fluorescence was recorded at the end of each annealing and extension step. All reactions were performed in duplicate and the starting quantity of the gene of interest was normalized to 18S rRNA for each sample. The delta-delta Ct method [48] was used to analyze alterations in gene expression and values were expressed as fold changes relative to control.

### Glutathione and glutathione disulfide levels

Levels of glutathione (GSH) and glutathione disulfide (GSSG) were determined using commercially available assay kits (Cayman Chemical, Ann Arbor, Michigan). Briefly, frozen TA tissues were thawed and homogenized in 10 volumes of buffer containing: 50 mM HEPES (pH 7.5), 150 mM NaCl, 1.5 mM MgCl_2_, 1 mM EDTA, 100 mM NaF, 10% glycerol, and 1% Nonidet P-40. To avoid potential interference due to particulates and sulfhydryl groups on proteins, samples were deproteinated with equal volumes of metaphosphoric acid, centrifuged at 5000g for 5 minutes, and supernatants mixed with triethanolamine (1:20 dilution). Aliquots of deproteinated samples were derivitized with 1M 2-vinylpyridine (1:100 dilution) for exclusive measurements of GSSG. Sulfhydryl groups were reacted with 5,5’-dithio-bis-2(nitrobenzoic acid) and the provided assay cocktail to produce 5-thio-2-nitrobenzoic acid. The absorbance at 405 nm was measured in duplicate after 25 min on a HTS 7000 Plus BioAssay plate reader (PerkinElmer, Waltham, Massachusetts). Final concentrations of GSH and GSSG (in μM) were calculated according to the manufacturer’s instructions.

### Total protein oxidation

Total protein oxidation was analyzed with the OxyBlot Protein Oxidation Detection Kit (Chemicon, Temecula, CA) according to the manufacturer’s directions. Briefly, TA muscles were homogenized in buffer containing 250 mM sucrose, 5 mM EDTA, 100 mM KCl, 20 mM Hepes, 2% β-mercaptoethanol and complete, mini protease inhibitor cocktail (Sigma). Carbonyl groups attached to the side chains of these protein lysates were derivitized to 2,4 dinitrophenylhydrazone and detected by dot blot analysis. Densitometry was performed using a Chemidoc XRS system and analyzed with Quantity One software (Biorad, Hercules, CA). Samples were run in duplicate and total protein oxidation was expressed as fold change relative to controls.

### Statistics

Comparison between control and alcohol groups after organized according to time post-injury were calculated with unpaired students’ t-tests using SigmaStat v2.0 software. One way analyses of variance followed by Student-Newman-Keuls post-hoc tests were performed when comparisons across multiple groups and times were required. Significance was accepted at p ≤ 0.05.

## Abbreviations

BaCl2: Barium chloride
CNTF: Ciliary neurotrophic factor
EtOH: Ethanol
GSH: Reduced glutathione
GSSG: Glutathione disulfide
IGF-1: Insulin-like growth factor-1
IL: Interleukin
NAC: N-acetyl cysteine
NSAIDS: Non-steroidal anti-inflammatory drugs
R.I.C.E.: Rest ice compression elevation
TA: Tibialis anterior
TGFβ1: Transforming growth factor β_1_
TNFα: Tumor necrosis factor α.

## References

[1] Smith C, Kruger MJ, Smith RM, Myburgh KH (2008). The inflammatory response to skeletal muscle injury: illuminating complexities. Sports Med.

[2] Prisk V, Huard J (2003). Muscle injuries and repair: the role of prostaglandins and inflammation. Histol Histopathol.

[3] Zhu J, Li Y, Lu A, Gharaibeh B, Ma J, Kobayashi T, Quintero AJ, Huard J (2011). Follistatin Improves Skeletal Muscle Healing after Injury and Disease through an Interaction with Muscle Regeneration, Angiogenesis, and Fibrosis. Am J Pathol.

[4] Bondesen BA, Mills ST, Kegley KM, Pavlath GK (2004). The COX-2 pathway is essential during early stages of skeletal muscle regeneration. American Journal of Physiology - Cell Physiology.

[5] Castets P, Bertrand AT, Beuvin M, Ferry A, Le Grand F, Castets M, Chazot G, Rederstorff M, Krol A, Lescure A (2011). Satellite cell loss and impaired muscle regeneration in selenoprotein N deficiency. Hum Mol Genet.

[6] Nguyen MH, Cheng M, Koh TJ (2011). Impaired muscle regeneration in ob/ob and db/db mice. ScientificWorldJournal.

[7] Otis JS, Brown LA, Guidot DM (2007). Oxidant-induced atrogin-1 and transforming growth factor-beta1 precede alcohol-related myopathy in rats. Muscle Nerve.

[8] Otis JS, Guidot DM (2009). Procysteine stimulates expression of key anabolic factors and reduces plantaris atrophy in alcohol-fed rats. Alcohol Clin Exp Res.

[9] Preedy VR, Peters TJ, Patel VB, Miell JP (1994). Chronic alcoholic myopathy: transcription and translational alterations. FASEB Journal.

[10] Lang CH, Fan J, Lipton BP, Potter BJ, McDonough KH (1998). Modulation of the insulin-like growth factor system by chronic alcohol feeding. Alcohol Clin Exp Res.

[11] Lang CH, Kimball SR, Frost RA, Vary TC (2001). Alcohol myopathy: impairment of protein synthesis and translation initiation. Int J Biochem Cell Biol.

[12] Hingson R, Howland J (1987). Alcohol as a risk factor for injury or death resulting from accidental falls: a review of the literature. J Stud Alcohol.

[13] Hingson R, Heeren T, Zakocs R, Winter M, Wechsler H (2003). Age of first intoxication, heavy drinking, driving after drinking and risk of unintentional injury among U.S. college students. J Stud Alcohol.

[14] Lang CH, Wu D, Frost RA, Jefferson LS, Kimball SR, Vary TC (1999). Inhibition of muscle protein synthesis by alcohol is associated with modulation of eIF2B and eIF4E. Am J Physiol.

[15] Lang CH, Frost RA, Deshpande N, Kumar V, Vary TC, Jefferson LS, Kimball SR (2003). Alcohol impairs leucine-mediated phosphorylation of 4E-BP1, S6K1, eIF4G, and mTOR in skeletal muscle. Am J Physiol Endocrinol Metab.

[16] Lang CH, Frost RA, Svanberg E, Vary TC (2004). IGF-I/IGFBP-3 ameliorates alterations in protein synthesis, eIF4E availability, and myostatin in alcohol-fed rats. Am J Physiol Endocrinol Metab.

[17] Fernandez-Sola J, Nicolas JM, Fatjo F, Garcia G, Sacanella E, Estruch R, Tobias E, Badia E, Urbano-Marquez A (2003). Evidence of apoptosis in chronic alcoholic skeletal myopathy. Hum Pathol.

[18] Preedy VR, Salisbury JR, Peters TJ (1994). Alcoholic muscle disease: features and mechanisms. Journal of Pathology.

[19] Garriga J, Adanero E, Fernandez-Sola J, Urbano-Marquez A, Cusso R (2000). Ethanol inhibits skeletal muscle cell proliferation and delays its differentiation in cell culture. Alcohol Alcohol.

[20] Garriga J, Fernandez-Sola J, Adanero E, Urbano-Marquez A, Cusso R (2005). Metabolic effects of ethanol on primary cell cultures of rat skeletal muscle. Alcohol.

[21] Hong-Brown LQ, Frost RA, Lang CH (2001). Alcohol impairs protein synthesis and degradation in cultured skeletal muscle cells. Alcohol Clin Exp Res.

[22] Otis JS, Guidot DM (2010). Procysteine increases alcohol-depleted glutathione stores in rat plantaris following a period of abstinence. Alcohol Alcohol.

[23] Zhu J, Li Y, Shen W, Qiao C, Ambrosio F, Lavasani M, Nozaki M, Branca MF, Huard J (2007). Relationships between transforming growth factor-beta1, myostatin, and decorin: implications for skeletal muscle fibrosis. J Biol Chem.

[24] Kami K, Morikawa Y, Sekimoto M, Senba E (2000). Gene expression of receptors for IL-6, LIF, and CNTF in regenerating skeletal muscles. J Histochem Cytochem.

[25] Clary CR, Guidot DM, Bratina MA, Otis JS (2011). Chronic alcohol ingestion exacerbates skeletal muscle myopathy in HIV-1 transgenic rats. AIDS Res Ther.

[26] Marques MJ, Neto HS (1997). Ciliary neurotrophic factor stimulates in vivo myotube formation in mice. Neurosci Lett.

[27] Kellett J (1986). Acute soft tissue injuries–a review of the literature. Med Sci Sports Exerc.

[28] McBrier NM, Lekan JM, Druhan LJ, Devor ST, Merrick MA (2007). Therapeutic ultrasound decreases mechano-growth factor messenger ribonucleic acid expression after muscle contusion injury. Arch Phys Med Rehabil.

[29] Mackey AL, Mikkelsen UR, Magnusson SP, Kjaer M (2012). Rehabilitation of muscle after injury - the role of anti-inflammatory drugs. Scand J Med Sci Sports.

[30] Velloso CP (2008). Regulation of muscle mass by growth hormone and IGF-I. Br J Pharmacol.

[31] Nozaki M, Li Y, Zhu J, Ambrosio F, Uehara K, Fu FH, Huard J (2008). Improved muscle healing after contusion injury by the inhibitory effect of suramin on myostatin, a negative regulator of muscle growth. Am J Sports Med.

[32] Chan YS, Li Y, Foster W, Fu FH, Huard J (2005). The use of suramin, an antifibrotic agent, to improve muscle recovery after strain injury. Am J Sports Med.

[33] Childs A, Jacobs C, Kaminski T, Halliwell B, Leeuwenburgh C (2001). Supplementation with vitamin C and N-acetyl-cysteine increases oxidative stress in humans after an acute muscle injury induced by eccentric exercise. Free Radic Biol Med.

[34] Vercherat C, Chung TK, Yalcin S, Gulbagci N, Gopinadhan S, Ghaffari S, Taneja R (2009). Stra13 regulates oxidative stress mediated skeletal muscle degeneration. Hum Mol Genet.

[35] Beaton LJ, Allan DA, Tarnopolsky MA, Tiidus PM, Phillips SM (2002). Contraction-induced muscle damage is unaffected by vitamin E supplementation. Med Sci Sports Exerc.

[36] Wang J, Chu H, Zhao H, Cheng X, Liu Y, Jin W, Zhao J, Liu B, Ding Y, Ma H (2007). Nitricoxide synthase-induced oxidative stress in prolonged alcoholic myopathies of rats. Mol Cell Biochem.

[37] Fernandez-Sola J, Preedy VR, Lang CH, Gonzalez-Reimers E, Arno M, Lin JC, Wiseman H, Zhou S, Emery PW, Nakahara T (2007). Molecular and cellular events in alcohol-induced muscle disease. Alcohol Clin Exp Res.

[38] Mantle D, Preedy VR (1999). Free radicals as mediators of alcohol toxicity. Adverse Drug Reactions & Toxicological Reviews.

[39] Preedy VR, Adachi J, Asano M, Koll M, Mantle D, Niemela O, Parkkila S, Paice AG, Peters T, Rajendram R (2002). Free radicals in alcoholic myopathy: indices of damage and preventive studies. Free Radical Biology & Medicine.

[40] Wang J, Liu Y, Zhang L, Ji J, Wang B, Jin W, Zhang C, Chu H (2012). Effects of increased matrix metalloproteinase-9 expression on skeletal muscle fibrosis in prolonged alcoholic myopathies of rats. Mol Med Report.

[41] Burks TN, Cohn RD (2011). Role of TGF-β signaling in inherited and acquired myopathies. Skelet Muscle.

[42] Curtis R, Adryan KM, Zhu Y, Harkness PJ, Lindsay RM, DiStefano PS (1993). Retrograde axonal transport of ciliary neurotrophic factor is increased by peripheral nerve injury. Nature.

[43] Ulenkate HJ, Kaal EC, Gispen WH, Jennekens FG (1994). Ciliary neurotrophic factor improves muscle fibre reinnervation after facial nerve crush in young rats. Acta Neuropathol.

[44] Wagner MC, Yeligar SM, Brown LA, Michael Hart C (2012). PPARγ ligands regulate NADPH oxidase, eNOS, and barrier function in the lung following chronic alcohol ingestion. Alcohol Clin Exp Res.

[45] O'Connor RS, Mills ST, Jones KA, Ho SN, Pavlath GK (2007). A combinatorial role for NFAT5 in both myoblast migration and differentiation during skeletal muscle myogenesis. J Cell Sci.

[46] Otis JS, Ashikhmin YI, Brown LA, Guidot DM (2008). Effect of HIV-1-related protein expression on cardiac and skeletal muscles from transgenic rats. AIDS Res Ther.

[47] Bradford MM (1976). A rapid and sensitive method for the quantitation of microgram quantities of protein utilizing the principle of protein-dye binding. Anal Biochem.

[48] Livak KJ, Schmittgen TD (2001). Analysis of relative gene expression data using real-time quantitative PCR and the 2(-Delta Delta C(T)) Method. Methods.

